# BMI Association With Treatment Outcomes in Head and Neck Cancer Patients Receiving Immunotherapy: A Comprehensive Review and Meta‐Analysis

**DOI:** 10.1002/cnr2.70147

**Published:** 2025-02-11

**Authors:** Sakditad Saowapa, Natchaya Polpichai, Pharit Siladech, Chalothorn Wannaphut, Manasawee Tanariyakul, Phuuwadith Wattanachayakul, Diego Olavarria Bernal, Hector Garcia Pleitez, Lukman Tijani

**Affiliations:** ^1^ Department of Internal Medicine Texas Tech University Health Sciences Center Lubbock Texas USA; ^2^ Department of Internal Medicine Weiss Memorial Hospital Chicago Illinois USA; ^3^ Department of Internal Medicine, Faculty of Medicine Ramathibodi Hospital Mahidol University Bangkok Thailand; ^4^ Department of Internal Medicine, John A. Burns School of Medicine University of Hawaii Honolulu Hawaii USA; ^5^ Department of Internal Medicine Einstein Medical Center Philadelphia Philadelphia Pennsylvania USA; ^6^ Hematology and Oncology Department Texas Tech University Health Sciences Center Lubbock Texas USA

**Keywords:** adverse effect, colitis, colon cancer, immunotherapy, metastatic colon cancer, side effects

## Abstract

**Background:**

In recent years, immunotherapy using immune checkpoint inhibitors (ICIs) has revolutionized the treatment of advanced malignancies. As such, numerous ICIs are establishing themselves as prospective therapy alternatives for individuals with head and neck cancer (HNC). Evidence suggests a potential correlation between body mass index (BMI) and the efficacy of ICIs in cancer patients. However, this association in HNC patients subjected to immunotherapy is still unclear.

**Aims:**

To investigate the effect of BMI on the survival outcomes of HNC patients treated with immunotherapy.

**Methods:**

PubMed, Web of Science, and Google Scholar databases were searched extensively for records published until January 2024. Full‐text articles aligned with the research objective were included, while records published in English, case reports, reviews, editorials, and studies reporting immunotherapy combined with other cancer therapies were excluded. The data required for review and analysis was abstracted in Excel files by two independent reviewers. Additionally, data synthesis was carried out using the Review Manager program, and evaluation of methodological quality was done with the Newcastle Ottawa scale. The statistical analyses were stratified according to the BMI values, of which patients were categorized as follows: Obese (BMI ≥ 27.5), non‐obese (BMI < 27.5), overweight (BMI: 23.5–27.5), underweight (BMI < 18.5), normal (BMI: 18.5–23.5), low (BMI < 20), and high (BMI ≥ 20).

**Results:**

Only six studies were reviewed and analyzed. A subgroup analysis of data from these studies showed that obese HNC patients on immunotherapy had significantly better overall survival (OS) rates than non‐obese patients (HR: 0.51; 95% CI: 0.29–0.93; *p* = 0.03). However, the progression‐free survival (PFS) was statistically similar between obese and non‐obese patients (HR: 0.72; 95% CI: 0.39–1.33; *p* = 0.30). In addition, when BMI was stratified as either low or high, no significant difference was observed in the OS and PFS of HNC patients (HR: 0.99; 95% CI: 0.59–1.66; *p* = 0.97 and HR: 0.93; 95% CI: 0.61–1.41; *p* = 0.42, respectively). Similarly, the statistical analyses showed that overweight patients have similar OS and PFS as patients with normal BMI (HR: 0.53; 95% CI: 0.15–1.92; *p* = 0.33 and HR: 0.55; 95% CI: 0.20–1.52; *p* = 0.25, respectively). In contrast, underweight patients demonstrated poor OS and PFS (HR: 2.56; 95% CI: 1.29–5.12; *p* = 0.008 and HR: 2.76; 95% CI: 1.17–6.52; *p* = 0.02, respectively).

**Discussion and Conclusion:**

Obese HNC patients on immunotherapy tend to have improved OS than non‐obese patients, while underweight patients have worse clinical prognoses than those with normal or above BMI.

## 1 Introduction

Head and neck cancer (HNC) is ranked the eighth most frequent cancer across the globe [1]. Despite breakthroughs in cancer treatments such as surgery, radiation therapy, and chemotherapy, HNC continues to be an enormous cause of premature death and morbidity globally [2, 3]. However, the introduction of immunotherapy, notably immune checkpoint inhibitors (ICIs) targeting programmed cell death 1 (PD‐1) and programmed cell death ligand 1 (PD‐L1), has altered the therapeutic landscape for advanced or metastatic (R/M) HNC. ICIs have shown considerable efficacy in some patients, resulting in durable responses and improved survival outcomes. For instance, the KEYNOTE‐40 trial showed that pembrolizumab, a humanized monoclonal antibody targeting PD‐1, improved the median overall survival (OS) duration and, therefore, deemed superior to traditional treatment as the second‐line therapy for R/M head and neck squamous cell carcinoma (HNSCC) [4]. Similarly, a recent clinical study demonstrated that nivolumab was linked with better OS than conventional treatment in platinum‐refractory R/M HNSCC patients [5]. Therefore, the investigators of that study suggested that nivolumab might serve as the conventional subsequent‐line therapy for R/M HNSCC. Nonetheless, a sizeable percentage of patients fail to derive substantial benefits from immunotherapy, calling for more inquiry into factors that impact treatment response and prognosis.

Recently, body mass index (BMI), an established marker for patients' nutritional health, has attracted great interest in cancer research. High BMI is steadily being correlated to deteriorating survival outcomes and greater risk of any grade immune‐related adverse events (IRAEs) in cancer patients receiving immunotherapy [6, 7, 8]. In contrast, high BMI, specifically obesity and overweight, has been associated with improved survival outcomes in HNC patients [9, 10]. However, there is minimal information on whether BMI influences the survival of HNC patients currently on immunotherapy. Therefore, the current systematic review was conducted to determine whether BMI has any influence on the survival outcomes of HNC patients subjected to immunotherapy.

## 2 Methodology

### 2.1 Information Sources and Searches

An in‐depth search for articles published up to January 2024 was conducted on PubMed, Web of Science, and Google Scholar databases. Furthermore, bibliographies of articles related to our topic were scrutinized for additional studies. The search strategy employed in the electronic databases to identify studies was as follows (Head and neck cancer OR head and neck squamous cell carcinoma OR squamous cell carcinoma OR HNSCC) AND (body mass index OR BMI OR nutritional status OR nutritional indices OR obesity OR underweight OR overweight) AND (immunotherapy OR immune check‐point inhibitors OR ICIs OR anti‐programmed cell death receptor‐1 OR PD‐1 OR anti‐programmed cell death ligand receptor‐1 OR PDL‐1 OR nivolumab OR pembrolizumab OR Atezolizumab OR anti‐cytotoxic T‐lymphocyte‐associated molecule‐4 OR ipilimumab OR Tremelimumab). Additionally, duplicate articles and grey literature were eliminated from the search as they would have interfered with the scientific purpose of the current research and undermined the statistical analyses.

### 2.2 Eligibility Criteria

Two reviewers independently screened full‐text articles retrieved from the electronic databases and included those that met the objective of the current study, that is, studies including HNC patients subjected to immunotherapy and reporting the role of BMI on survival outcomes. Furthermore, only scientific research journals published in English were considered. Conversely, papers that did not correspond with the inclusion criteria or those designed as case reports, on‐going clinical studies, or reviews were omitted. In addition, papers describing the role of BMI in predicting the survival outcomes of HNC patients subjected to immunotherapy coupled with other cancer therapies were eliminated.

### 2.3 Data Extraction and Evaluated Outcomes

Two impartial reviewers assessed full‐text articles of included studies and abstracted the data required for review and analysis into separate Excel files. Afterward, the data was harmonized into a single characteristic table. The data collected by the reviewers included Author ID, study design, the country where the research was done, pertinent characteristics of HNC patients treated with immunotherapy (sample size and sex distribution), the immunotherapy regimen administered, BMI stratification, and survival outcomes evaluated. In the event of discrepancies in the extracted data, the two reviewers engaged in constructive discussion, and if they could not reach a compromise, an additional reviewer was contacted.

The primary outcomes of our study were overall survival (OS) and progression‐free survival (PFS). OS has been interpreted as the period from the commencement of immunotherapy to mortality due to any cause. On the other hand, PFS was described as the period from the first dose of immunotherapy to progression of the disease or all‐cause mortality.

### 2.4 Quality Appraisal

All the included studies were observational; therefore, methodological quality evaluation was undertaken with the Newcastle Ottawa Scale (NOS). Using this technique, studies were graded based on the selection, comparability, and outcome domains. For each category, a maximum of one star was awarded for a completely answered criterion; otherwise, no star was assigned. Within the selection category, an aggregate of 4 stars could be obtained, while a ceiling of two and three stars could be attained for the comparability and outcome domains, respectively. Furthermore, the overall study quality was evaluated by converting the NOS scores to AHRQ standards.

### 2.5 Data Synthesis

Data synthesis was carried out using the Review Manager software (RevMan version 5.4.1) to provide valuable insights into the correlation between BMI and survival outcomes (i.e., OS and PFS) of HNC patients subjected to immunotherapy. The DerSimonian‐Laird random effects model was utilized throughout the research to offset all the predicted variability and produce conservative estimations. Furthermore, the statistical variability between the eligible studies was examined via the I2 statistics, of which values higher than 50% were deemed substantial. The overall effect size was assessed using hazard ratios (HR) and their respective 95% confidence intervals (CIs), of which an overall HR with a *p*‐value of less than 0.05 indicated a significant association between BMI and OS or PFS. Subgroup analyses depending on the BMI stratification were conducted. The BMI stratification was made as follows: Obese (BMI ≥ 27.5), non‐obese (BMI < 27.5), overweight (BMI: 23.5–27.5), underweight (BMI < 18.5), normal (BMI: 18.5–23.5), low (BMI < 20), and high (BMI ≥ 20).

## 3 Results

### 3.1 Study Selection

**FIGURE 1:**
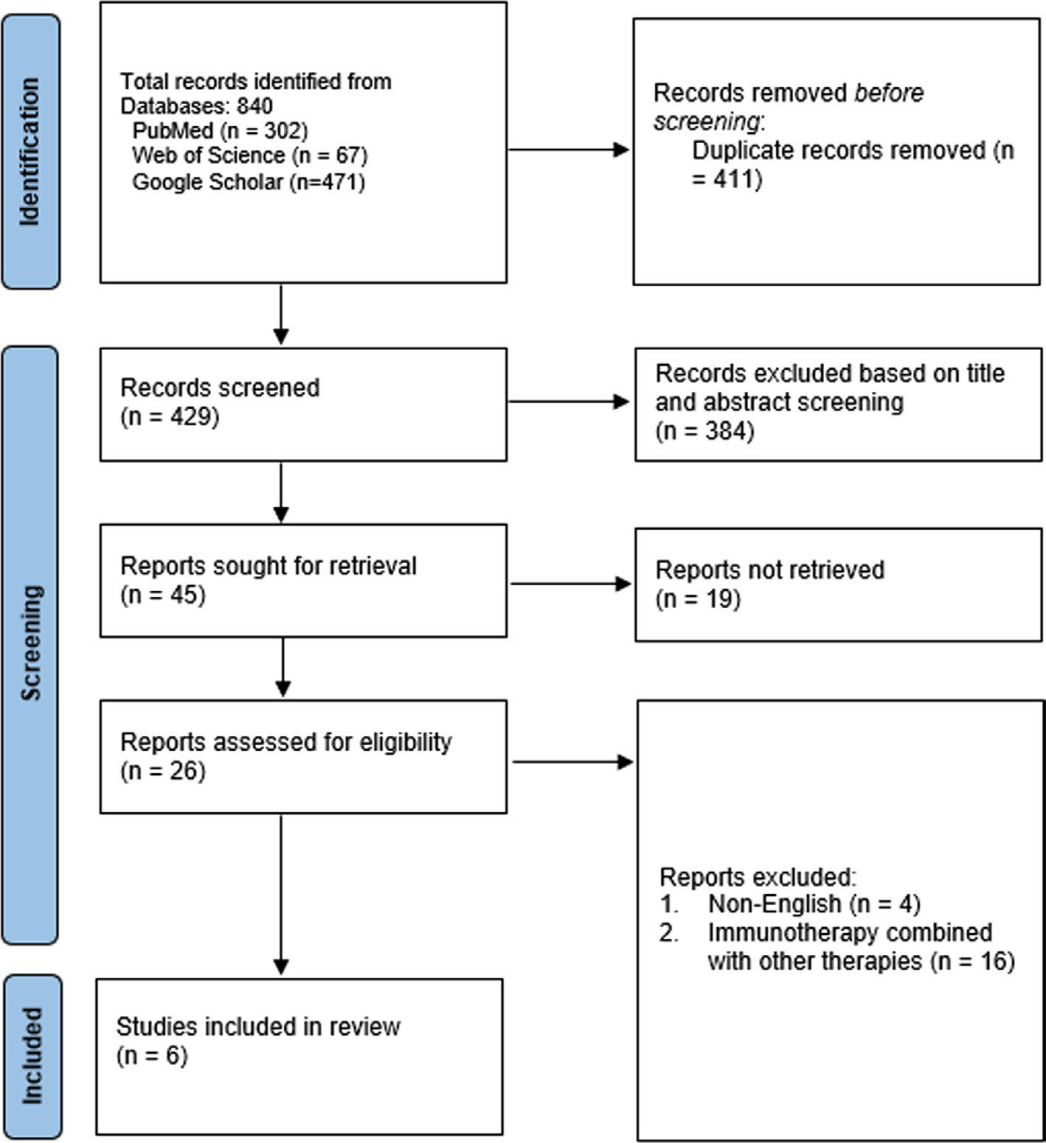
PRISMA flow diagram for study selection.

A preliminary query to the electronic databases resulted in 840 articles containing the specified MeSH phrases. A rigorous duplicate investigation of these articles resulted in the elimination of 411 articles considered close or exact duplicates. Out of the remaining 429 papers, 384 with irrelevant titles and abstracts were eliminated. Moreover, 19 research articles were not retrieved since they were designed as case reports, conference abstracts, review articles, or editorials. Finally, only 6 publications qualified for evaluation and analysis, while the remaining 20 articles were eliminated due to the reasons listed below: 4 were published in other languages, and 16 comprised patients subjected to immunotherapy alongside other cancer treatments. The complete selection criteria and number included studies are shown in Figure 1.

### 3.2 Study Characteristics

A total of 454 HNC patients subjected to immunotherapy were analyzed in our meta‐analysis. Regarding the immunotherapy administered, pembrolizumab was reported in one study [11], nivolumab in two studies [12, 13], nivolumab or pembrolizumab in two studies [14, 15], and PD‐1/PDL‐1 monotherapies or combinations with anti‐CTLA‐4 in one study [16]. According to the BMI stratification, one study [15] stratified BMI values into high and low, with a cut‐off value of 20, two studies [13, 14] stratified BMI according to the World Health Organization (WHO) guidelines for the general population, two studies [11, 12] stratified BMI values according to the established cut‐off values for Asian population, and one study stratified BMI values as either obese (BMI ≥ 30) or non‐obese (BMI < 30) [16]. These study characteristics are summarized in Table 1.

**TABLE 1:** Summary of study characteristics.

Author ID	Study design	Country	Characteristics of HNC patients treated with immunotherapy		Immunotherapy regimen	BMI stratification	Outcomes
			Sample (*n*)	M/F			
Zhang et al. [11]	Retrospective cohort study	China	49	14/35	Pembrolizumab	Normal: 18.5–23.5; Obese: ≥ 27.5; Overweight: 23.5–27.5; underweight: < 18.5	OS and PFS
Chikuie et al. [12]	Retrospective cohort study	Japan	56	40/16	Nivolumab	Normal: 18.5–23.5; underweight: < 18.5; above: ≥ 25	OS and PFS
Arihara et al. [13]	Retrospective cohort study	Japan	44	38/6	Nivolumab	Normal: 18.5–25; underweight: < 18.5; overweight/obese: ≥ 25	OS
Guller et al. [14]	Retrospective cohort study	United States	99	86/13	Nivolumab or Pembrolizumab	Normal: 18.5–24.9; overweight 25–29.9; obese: ≥ 30	OS and PFS
Miyamoto et al. [15]	Retrospective cohort study	Japan	106	83/23	Nivolumab or Pembrolizumab	Low: < 20; High: ≥ 20	OS and PFS
Hernando‐Calvo et al. [16]	Retrospective cohort study	Spain	100	82/18	PD‐1/PDL‐1 monotherapies or combinations with anti‐CTLA‐4	Obese: ≥ 30; Others: < 30	OS and PFS

Abbreviations: anti‐CTLA‐4, anti‐cytotoxic T‐lymphocyte associated protein 4; BMI, body mass index; HNC, head and neck cancer; OS, overall survival; PFS: progression‐free survival; PD‐1, programmed cell death 1; PD‐L1, programmed cell death ligand 1.

### 3.3 Quality Evaluation Outcomes

Our assessment has shown that all studies had good methodological quality. None of the studies was able to attain a maximum score in the selection domain because they were carried out in single centers, which may not represent the general population (Table 2).

**TABLE 2:** Methodological quality assessment using the Newcastle Ottawa Scale.

Author ID	Selection of participants	Comparability	Reporting of outcomes	AHRQ quality
Zhang et al. [9]	3	1	3	Good
Chikuie et al. [10]	3	1	3	Good
Arihara et al. [11]	3	1	3	Good
Guller et al. [12]	3	1	3	Good
Miyamoto et al. [13]	3	1	3	Good
Hernando‐Calvo et al. [14]	3	1	3	Good

### 3.4 Association Between Body Mass Index (BMI) and Overall Survival (OS)

**FIGURE 2:**
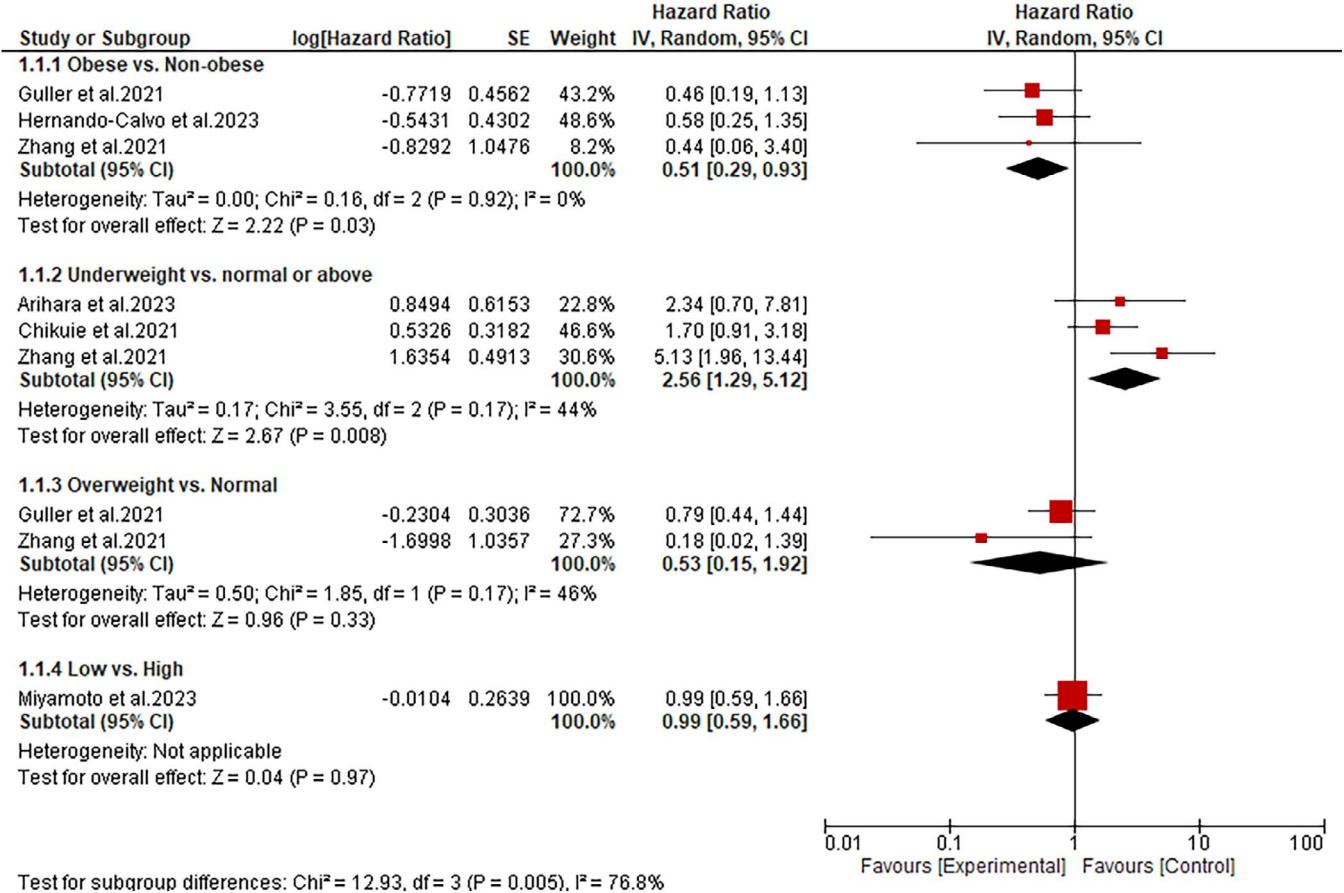
A forest plot showing the association between BMI and OS.

The overall effect of BMI on OS was evaluated in all included studies. A subgroup analysis of data from these studies showed that obese HNC patients treated with immunotherapy have significantly improved OS than non‐obese patients (HR: 0.51; 95% CI: 0.29–0.93; *p* = 0.03) (Figure 2). Additionally, the subgroup analysis showed a substantial improvement in OS among HNC normal‐weight patients than underweight patients (HR: 2.56; 95% CI: 1.29–5.12; *p* = 0.008) (Figure 2). On the contrary, compared to overweight HNC patients, those with normal BMI had similar improvements in OS after being treated with immunotherapy (HR: 0.53; 95% CI: 0.15–1.92; *p* = 0.33). Similarly, we found that HNC patients with high BMI (> 20) have a statistically similar improvement in OS as patients with low BMI (≤ 20) (HR: 0.99; 95% CI: 0.59–1.66; *p* = 0.97) (Figure 2).

### 3.5 Association Between BMI and PFS

**FIGURE 3:**
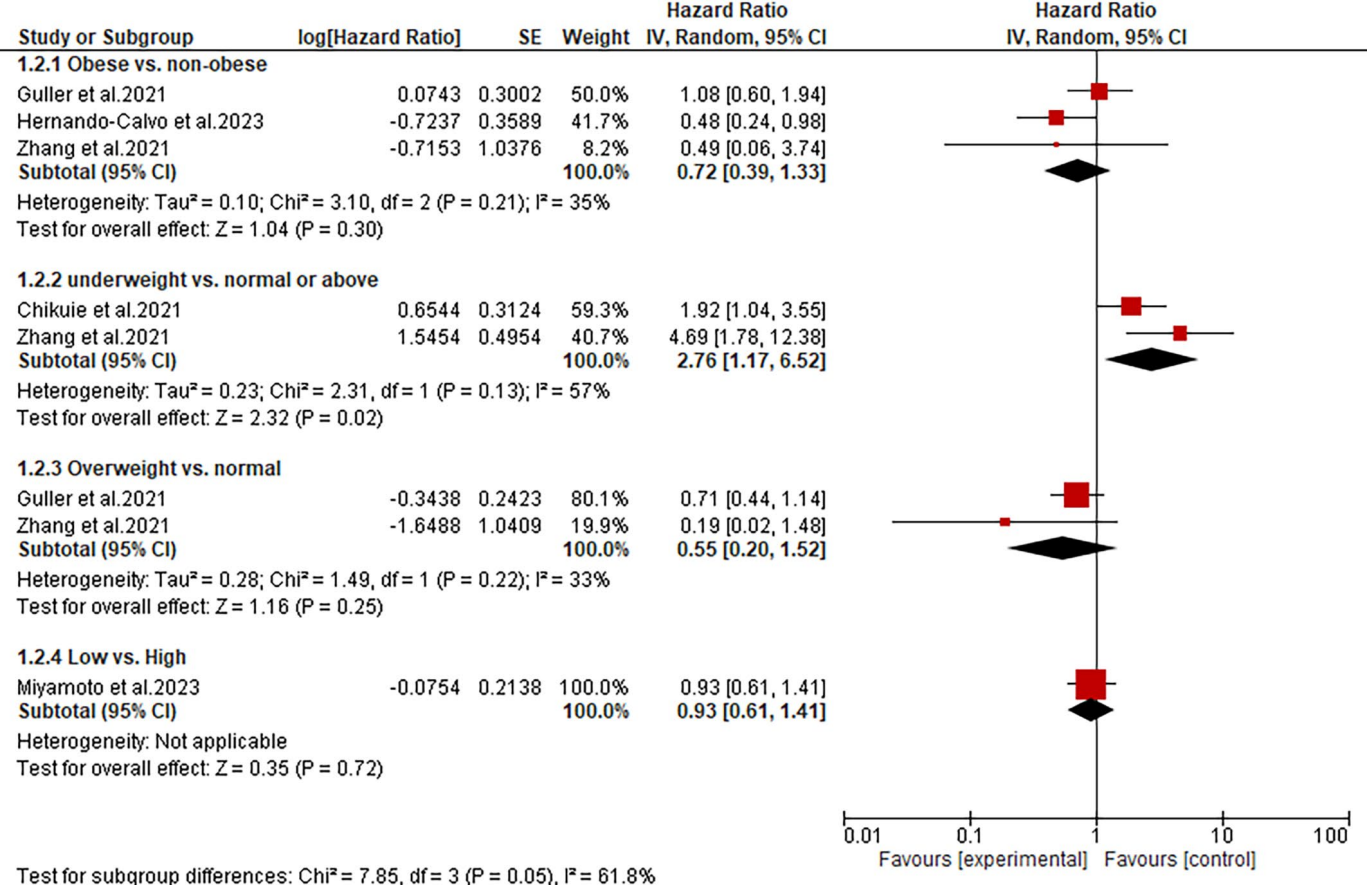
A forest plot showing the association between BMI and PFS.

Subgroup analyses performed according to the BMI values revealed that underweight HNC patients treated with immunotherapy have significantly poor PFS than those with normal/above BMI values (HR: 2.76; 95% CI: 1.17–6.52; *p* = 0.02) (Figure 3). Conversely, the subgroup analysis demonstrated an insignificant difference in PFS between obese and non‐obese patients (HR: 0.72; 95% CI: 0.39–1.33; *p* = 0.30), overweight and normal BMI patients (HR: 0.55; 95% CI: 0.20–1.52; *p* = 0.25) and between low and high BMI (HR: 0.93; 95% CI: 0.61–1.41; *p* = 0.72) (Figure 3).

## 4 Discussion

Previous research articles have revealed that nutritional health strongly correlates with better survival outcomes following treatment of solid tumors with PD‐1 inhibitors [17]. However, this connection among HNC patients subjected to immunotherapy is limited. Therefore, we explored the causal relationship between BMI and survival outcomes in HNC patients who had received immunotherapy. In this respect, we discovered that BMI is an autonomous prognostic factor, as HNC obese patients receiving immunotherapy tend to have better OS than non‐obese patients, while underweight patients tend to have worse OS and PFS.

It is paradoxical that despite obesity being known to elevate the likelihood of developing cancer, it improved OS in HNC patients subjected to immunotherapy. This finding, however, corresponds with what has been observed in past studies. For instance, a recent retrospective analysis indicated that compared to normal‐weight melanoma patients, obese patients given ICIs had superior PFS and OS [18]. Similarly, a meta‐analysis of 5279 cancer patients receiving ICI treatments found a favorable correlation between obesity and improved OS [19]. Although the specific mechanism by which BMI influences survival outcomes following the administration of immunotherapy is unclear, the plausible theory could be that obesity increases the creation of a systemic meta‐inflammation, which results in irregular immune response. This is clear in a prior research indicating that adipocytes in adipose tissues may produce various proinflammatory cytokines and chemokines to build and sustain the inflammatory milieu, which could intensify the action of ICIs [20]. Additionally, an experimental research indicated that obesity has the capacity to promote T cell malfunction and elevate the amount of PD‐1 positive T cells within the peripheral blood and tumors [21]; hence, this might partly explain the favorable impact of obesity on the effectiveness of ICIs. Furthermore, eating disorders are frequent in R/M HNSCC patients, and this could result in malnutrition and cachexia, two diseases that are closely connected with poor prognosis [22]. Therefore, obese people utilize their body's food resources and hence may achieve a prolonged recovery with a more favorable prognosis after immunotherapy treatment.

Notably, we found that underweight HNC patients treated with immunotherapy tend to have poor OS and PFS than patients with normal/above BMI. This observation is comparable with findings in individuals with different malignancies receiving ICIs [17, 23]. Therefore, being underweight is a potential biomarker for poor response to immunotherapy, and thus, early nutritional intervention is of utmost importance in this population. Early nutritional therapies generally comprise oral consumption of vitamins or feeding via gastrointestinal tube to make up for the poor oral intake [24]. Despite underweight being associated with poor prognosis, our subgroup has shown that when the cut‐off BMI value is 20, HNC patients with low BMI have similar PFS and OS as patients with high BMI. One possible explanation for this disparity is that some patients with normal BMI values were also used in the same analysis as those with low BMI. Therefore, we believe that the prognostic value of BMI in cancer patients subjected to immunotherapy should be analyzed based on the BMI stratification advocated by the WHO.

Contrary to previous literature, our study showed no difference in OS and PFS between normal and overweight patients. These contradictory results might be explained by the fact that our study only assessed evidence from patients with HNC, while the other studies involved patients with different cancer types. Another contributing factor to the inconsistent results is that only two studies with small sample sizes were available for the survival analyses. Therefore, more research on the association between overweight and survival of HNC patients treated with immunotherapy is required to support our findings.

While our study has shown a favorable correlation between obesity and improved prognosis for HNC patients undergoing ICI treatment, the absence of a uniform cut‐off BMI value might have affected our outcomes. The current review used a cut‐off BMI value of 27.5 kg/m^2^ to indicate obesity. This cut‐off value was mainly chosen because, unlike the general population, the suitable BMI value for obesity in the Asian population is 27.5 kg/m^2^ [25]. However, according to the WHO guidelines, the cut‐off value for obesity is 30 kg/m^2^ [26]. This means that patients deemed overweight according to WHO guidelines were used in the analysis of obesity and vice versa. Thus, we suggest that future research look into establishing a universal BMI cut‐off value in order to better understand how BMI affects the clinical results of ICI‐treated cancer patients.

The correlation between BMI and IRAEs is also documented in several studies. It should be noted that the correlation between BMI and IRAEs in malignancy patients taking ICIs appears to be linear. Cortellini and colleagues reported that among cancer patients subjected to PD‐1/PD‐L1 checkpoint inhibitors, obesity was the only clinically significant factor associated with grade 3–4 IRAEs and IRAEs prompting cessation of treatment [8]. Specifically, obese individuals had a higher prevalence of more clinically important IRAEs (i.e., gastrointestinal, hepatic, and pulmonary), that might be disproportionately associated with better clinical outcomes. Furthermore, obese individuals are the only group that had higher occurrences of rheumatic IRAEs. On the contrary, it was found that overweight individuals had the highest frequency of cutaneous and endocrine IRAEs. Unfortunately, these findings could not be confirmed in a retrospective cohort study assessing the relationship between BMI and the efficacy of pembrolizumab, a second‐line therapy for patients with R/M HNSCC. Specifically, the study found the occurrence of any grade IRAEs to be statistically similar between underweight, normal, overweight, and obese patients (*p* = 0.375). This minor difference resonated with the exceptionally small number of enrolled patients in the trial and the absence of follow‐up for every immunotherapy. In a similar fashion, a meta‐analysis of three studies did not find any difference in IRAE incidence between obese and normal cancer patients on ICIs, suggesting that there is no association between BMI and the incidence of IRAEs [19]. Therefore, further investigation is warranted to establish the role of BMI in the occurrence of IRAEs in tumor patients, specifically those with HNC on immunotherapy.

Although the current research has shown that BMI correlates with the prognosis of HNC patients on immunotherapy, it is not an appropriate approach to measure nutritional health, as indicated by the high BMI and low muscle mass in sarcopenic obese patients [27]. Therefore, other nutritional indices such as skeletal muscle index (SMI) and prognostic nutritional index (PNI) have been used as prognostic factors in malignancy patients subjected to immunotherapy. PNI is a nutritional index devised by Onodera and colleagues to predict postoperative complications [28]. This measure is generated from the concentration of serum albumin and the quantity of lymphocytes in the peripheral blood. The predictive value of PNI has been reported in patients with various cancers, including those with lung cancer, urothelial carcinoma, gastric cancer, and HNC [29, 30]. A previous meta‐analysis of nine studies showed that low PNI was linked with a low response rate and poor PFS and OS. These findings have also been replicated in HNC patients treated with ICIs. For instance, Hernando‐Calvo and colleagues found that PNI was the only nutritional index associated with PFS and OS in R/M HNSCC patients receiving immunotherapy [16]. Similarly, Guller and colleagues demonstrated that among patients with advanced HNC treated with anti‐PD‐1, anti‐CTLA‐4 antibodies, or both, PNI was significantly correlated to OS and PFS [14]. In contrast, Sakai et al. [30] reported that in patients with R/M HNSCC treated with ICIs, PNI was not predictive of OS and PFS. Although the exact reason for this discrepancy was not noted, it may have been partially caused by the use of different cut‐off values. Collectively, these findings suggest the need for high‐quality randomized trials to evaluate the efficacy of PNI in predicting clinical outcomes of cancer patients (especially those with HNSCC) treated with immunotherapy.

### 4.1 Implications for Clinical Care and Future Research

The results of this meta‐analysis might help oncologists consider BMI as an important factor for optimizing treatment plans. For instance, obese patients might be counseled differently regarding treatment expectations compared with underweight patients. Our results might also lead to the reconsideration of weight loss recommendations, that is healthcare providers might adjust their nutrition support guidelines by emphasizing maintaining or managing weight rather than aggressive weight loss during treatment with immunotherapy Moreover, BMI might be a useful predictive tool in clinical practice and can be a reliable stratification factor for prospective clinical trials of HNC patients treated with ICIs. Nonetheless, our findings were based on retrospective studies. Therefore, further research in large‐sample prospective studies is needed to support these findings. Furthermore, evidence suggests that BMI is not an appropriate measure of nutritional health. As such, we believe that it is vital for future studies to explore the prognostic significance of BMI alongside other nutritional and inflammatory markers. In addition, the lack of a uniform BMI cut‐off makes it challenging to ascertain the exact BMI value that significantly impacts survival. Therefore, future research should establish a universal BMI cut‐off value to better understand how BMI affects the clinical results of ICI‐treated cancer patients. Our results can also spark research into the reasons why obesity is associated with improved outcomes. This includes investigating metabolic, hormonal, and immune‐modulatory mechanisms in obese patients that result in improved outcomes. Therefore, opening pathways to new therapeutic targets and combination therapies.

### 4.2 Limitations

While interpreting the results of the current study, it is vital to keep in mind that it possesses some limitations. First, significant heterogeneity was observed in the subgroup assessing PFS between underweight and normal BMI patients. This heterogeneity might have resulted from the use of different ICI agents. However, the use of the random‐effects model was justified as we were able to provide more conservative effect sizes. Second, due to variations in cut‐off BMI values to stratify obese, overweight, and normal patients among the general and Asian population, it is possible that our meta‐analysis was influenced. Third, only studies published in English were eligible for review and analysis, meaning that our study was subject to reporting bias because data published in other languages was omitted. Fourth, we could not conduct a statistical synthesis to investigate the connection between BMI and IRAEs because only one included study evaluated this association. Therefore, additional research is necessary to evaluate the interaction of BMI and IRAEs in HNC patients exposed to immunotherapy and make a clear conclusion on the potential association. Fifth, none of the included studies reported the relationship between BMI and tumor response to immunotherapy. Therefore, future studies should investigate this potential association. Sixth, we strictly included patients treated with immunotherapy rather than those receiving immunotherapy concomitantly with other cancer treatments because we did not want those other treatments to influence our outcomes. However, in most cases patients had received prior cancer therapies, meaning our findings were subject to confounding bias. Finally, most studies in this review had small sample sizes, which might have been why we did not see any correlation between overweight and the survival of HNC patients on immunotherapy.

## 5 Conclusion

The present meta‐analysis has shown a strong correlation between BMI and survival outcomes of HNC patients undergoing treatment with immunotherapy. Specifically, obese HNC patients on immunotherapy have improved OS than non‐obese patients. On the other hand, compared to patients with normal/above BMI, underweight patients had a poor prognosis. Furthermore, research suggests that there is no substantial relationship between BMI and IRAEs in HNC patients undergoing immunotherapy. However, this finding still needs further investigation to make a firm conclusion.

## Author Contributions

**Sakditad Saowapa:** conceptualization, methodology, investigation, writing – original draft, writing – review and editing, project administration, supervision. **Natchaya Polpichai:** methodology, conceptualization, investigation, writing – original draft, writing – review and editing. **Pharit Siladech:** writing – original draft, writing – review and editing. **Chalothorn Wannaphut:** writing – original draft, writing – review and editing. **Manasawee Tanariyakul:** writing – original draft, writing – review and editing. **Phuuwadith Wattanachayakul:** writing – original draft, writing – review and editing. **Diego Olavarria Bernal:** writing – review and editing. **Hector Garcia Pleitez:** writing – review and editing. **Lukman Tijani:** methodology, investigation, writing – review and editing.

## Ethics Statement

The authors have nothing to report.

## Conflicts of Interest

The authors declare no conflicts of interest.

## Data Availability

The data that support the findings of this study are available on request from the corresponding author. The data are not publicly available due to privacy or ethical restrictions.
